# A systematic analysis of the role of GGDEF-EAL domain proteins in virulence and motility in *Xanthomonas oryzae* pv. *oryzicola*

**DOI:** 10.1038/srep23769

**Published:** 2016-04-07

**Authors:** Chao Wei, Wendi Jiang, Mengran Zhao, Junjie Ling, Xin Zeng, Jun Deng, Dongli Jin, John Maxwell Dow, Wenxian Sun

**Affiliations:** 1 Department of Plant Pathology and the Ministry of Agriculture Key Laboratory for Plant Pathology, China Agricultural University, Beijing, China; 2 School of Microbiology, BioSciences Institute, University College Cork, Cork, Ireland

## Abstract

The second messenger c-di-GMP is implicated in regulation of various aspects of the lifestyles and virulence of Gram-negative bacteria. Cyclic di-GMP is formed by diguanylate cyclases with a GGDEF domain and degraded by phosphodiesterases with either an EAL or HD-GYP domain. Proteins with tandem GGDEF-EAL domains occur in many bacteria, where they may be involved in c-di-GMP turnover or act as enzymatically-inactive c-di-GMP effectors. Here, we report a systematic study of the regulatory action of the eleven GGDEF-EAL proteins in *Xanthomonas oryzae* pv. *oryzicola*, an important rice pathogen causing bacterial leaf streak. Mutational analysis revealed that XOC_2335 and XOC_2393 positively regulate bacterial swimming motility, while XOC_2102, XOC_2393 and XOC_4190 negatively control sliding motility. The Δ*XOC_2335/XOC_2393* mutant that had a higher intracellular c-di-GMP level than the wild type and the Δ*XOC_4190* mutant exhibited reduced virulence to rice after pressure inoculation. *In vitro* purified XOC_4190 and XOC_2102 have little or no diguanylate cyclase or phosphodiesterase activity, which is consistent with unaltered c-di-GMP concentration in Δ*XOC_4190*. Nevertheless, both proteins can bind to c-di-GMP with high affinity, indicating a potential role as c-di-GMP effectors. Overall our findings advance understanding of c-di-GMP signaling and its links to virulence in an important rice pathogen.

Cyclic diguanylate (c-di-GMP) was initially discovered as an allosteric activator of cellulose synthesis in *Gluconacetobacter xylinus*^1^,^2^. The molecule is now recognized as an universal second messenger in bacteria that regulates a wide range of functions including cell differentiation, bacterial adhesion and biofilm formation, bacterial motility, colonization of host tissues and virulence^3^,^4^. The c-di-GMP-mediated signaling network is complex and regulation can occur at multiple levels to include transcription, by binding to transcription factors such as FleQ^5^, post-transcriptional, such as binding to GEMM RNAs^6^, and at the posttranslational level, such as in the regulation of Pel polysaccharide synthesis^7^,^8^. Cyclic di-GMP is formed from two GTP molecules by diguanylate cyclases (DGCs) that have a GGDEF domain and is broken into pGpG or GMP by phosphodiesterases (PDEs) containing either an EAL or HD-GYP domain^4^. These domains involved in c-di-GMP metabolism are widely present in Gram-negative bacterial proteins. For example, the *Escherichia coli* K-12 strain contains 29 GGDEF/EAL domain proteins, whereas *Vibrio cholerae* and *Pseudomonas aeruginosa* has 53 and 38 such proteins, respectively^9^,^10^. By contrast, the HD-GYP proteins are less common and even absent in some bacterial species^11^. These c-di-GMP metabolism proteins precisely modulate intracellular concentrations of c-di-GMP, and thus alter phenotypes through regulating different signaling pathways^12^.

A major sub-group of proteins involved in c-di-GMP signaling contain both GGDEF and EAL domains arranged in tandem^13^. Several such proteins have been demonstrated to have both DGC and PDE enzymatic activities; for example *Ms*DGC-1 in *Mycobacterium smegmatis*^14^, Lpl0329 in *Legionella pneumophila*^15^, and ScrC in *Vibrio parahemeolyticus*^16^. In many cases however, one of the two domains in the GGDEF-EAL proteins is catalytically inactive. For example, *Ax*DGC2 in *G. xylinus* only displays the DGC activity^17^, whereas CC3396, a GGDEF-EAL protein from *Caulobacter crescentus*, has only the PDE activity^18^. The inactive GGDEF or EAL domains of these proteins may act in a regulatory capacity. The DGC-inactive GEDEF domain of CC3396 is able to bind GTP and activates the PDE activity in the neighboring EAL domain^18^. The third situation is that both domains are enzymatically inactive but instead function as c-di-GMP effectors. For example, LapD from *Pseudomonas fluorescens* serves as a high affinity c-di-GMP receptor via a degenerate EAL domain and controls biofilm formation through regulating localization of the large cell surface adhesin LapA^19^,^20^. Another GGDEF-EAL protein Filp in *Xanthomonas oryzae* pv. *oryzae* (*Xoo*) also acts as the receptor of c-di-GMP that binds to the degenerate EAL domain with high affinity^21^. Mutation of the *filp* gene in *Xoo* attenuates bacterial virulence^21^. Recently, it was demonstrated in *Xylella fastidiosa* that a Tn*5* insertion mutant of *PD1671*, which encodes a putative GGDEF-EAL protein, has a hypervirulent phenotype in grapevines. This negative effect of PD1671 on virulence was attributed to enhanced expression of *gum* genes leading to increased production of the fastidian exopolysaccharide and associated biofilm formation^22^.

*X. oryzae* pv. *oryzicola* (*Xoc*) causes bacterial leaf streak (BLS) in rice, one of the most important bacterial diseases in subtropical Asia. BLS has expanded rapidly in South China and South-East Asia; no resistance genes to this disease are available^23^. Although BLS disease symptoms are very similar to those of another important rice disease, bacterial leaf blight caused by *Xoo*, the two causal pathogens have different infection processes and styles. *Xoc* initially enters leaf tissues of rice through stomata or wounds, and then colonizes the intercellular space of mesophyll while *Xoo* infects leaf through water pores and causes a systemic vascular disease^23^,^24^. Genome-wide mutational analyses have revealed multiple factors that contribute to *Xoc* virulence. These factors include type III secretion, lipopolysaccharide synthesis, type IV pilus and twitching motility, carbohydrate synthesis and two-component regulation^25^,^26^. As in other xanthomonads, c-di-GMP associated signalling pathways are also implicated in *Xoc* virulence. In *X. campestris* pv. *campestris*, multiple GGDEF/EAL/HD-GYP domain proteins have shown to contribute to virulence and environmental adaptation^27^. The HD-GYP domain regulator RpfG acts together with the sensor kinase RpfC in a two-component system to regulate the synthesis of particular virulence factors in response to the diffusible signal factor DSF^28^,^29^,^30^. Similarly, deletion of *rpfG* in *Xoc* results in reduced virulence^31^, suggesting an important role for c-di-GMP signaling. The *Xoc* BLS256 genome encodes 32 GGDEF/EAL/HD-GYP proteins with a potential role in c-di-GMP metabolism and perception^31^. No functional study on GGDEF and/or EAL domain-containing proteins in *Xoc* has been reported so far however.

In the present study, we constructed a panel of strains each with a deletion of one of the eleven genes that encode GGDEF-EAL proteins in *Xoc*. The effects of these mutations on virulence-associated phenotypes and virulence were systematically investigated. Four of these proteins (XOC_2102, XOC_2335, XOC_2393, and XOC_4190) influenced motility and one of them, XOC_4190, influenced virulence. We further demonstrated that *in vitro* purified XOC_4190 and XOC_2102 were enzymatically inactive, but were able to bind to c-di-GMP with high affinity. The findings add to an understanding of c-di-GMP signaling and its links to virulence in this important rice pathogen.

## Results

### A panel of deletion mutants for eleven genes encoding GGDEF-EAL domain-containing proteins in *Xoc*

The *Xoc* BLS256 genome encodes eleven tandem GGDEF-EAL domain-containing proteins^32^ (see Supplementary Fig. S1). Most of these proteins have additional sensory and signal transduction domains. Accordingly, XOC_1633, XOC_2102, XOC_2277 and XOC_2395 contain PAS domains that have been shown to sense diverse changes in environmental or cellular cues, such as light, redox state and oxygen^33^; XOC_2179, XOC_2277 and XOC_2466 carry GAF domains that in other proteins have been implicated in small ligand binding and protein-protein interactions^34^; XOC_2120 and XOC_2944 contain HAMP domains that may be associated with plasma membrane localization and signaling; XOC_2335 contains three novel conserved MHYT domains with a likely signaling function^35^; and XOC_2102, XOC_2393 and XOC_4190 have REC domains and may function as regulators in two-component systems^36^,^37^. These functional domains in the proteins indicate that their activities in cyclic di-GMP turnover or perception are responsive to environmental cues^9^,^12^. Deletion mutants were constructed for the eleven genes encoding these proteins following the strategy described in the Materials and Methods section and were listed in Supplementary Table S1. All mutants used for phenotypic studies were confirmed by Southern blot analyses (see Supplementary Fig. S2).

### XOC_2335 and XOC_ 2393 positively regulate swimming motility

**Figure 1:**
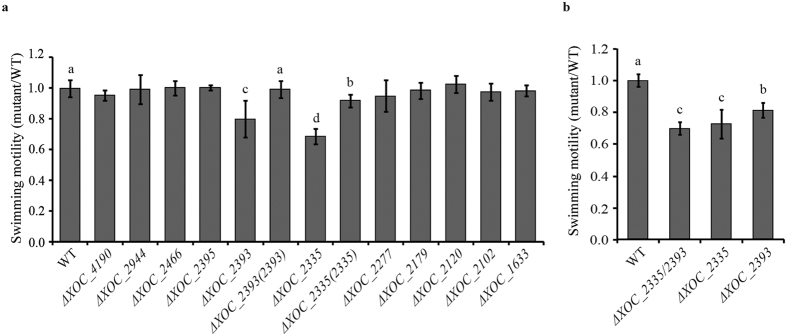
Effects of mutation of individual genes encoding GGDEF-EAL proteins on the swimming motility of *Xoc*. (**a**) The swimming motility of Δ*XOC_2335* and Δ*XOC_2393* was significantly reduced compared with that of the wild-type and other gene-deletion strains. The complementation strains Δ*XOC_2335(2335)* and Δ*XOC_2393(2393)* with plasmid-borne full-length genes restored the swimming motility nearly to or completely to the wild-type level, respectively. (**b**) The double-gene deletion mutant Δ*XOC_2335*/*XOC_2393* exhibited a similar swimming ability to the Δ*XOC_2335* mutant. The swimming motility of different *Xoc* strains was evaluated on semisolid plates with 0.2% noble agar after incubating at 28 °C for 4 d. The ratios of colony diameter of the mutant strains to the wild type were shown. Bars are means ± standard error (SE). The letters (a–d) indicate significant difference (*P* < 0.05) by Duncan’s multiple range test.

Swimming motility is a major survival mechanism of most Gram-negative bacteria. In bacteria, high level of c-di-GMP often suppresses swimming motility^11^. Therefore, the panel of mutants was first tested for swimming motility on semisolid (0.2% agar) medium plates. The results showed that the swimming motility of Δ*XOC_2335* and Δ*XOC_2393* was attenuated by ~30% and ~20% compared with the wild-type strain (Fig. 1a). By contrast, other mutants displayed swimming motility similar to the wild type (Fig. 1a). The complementation of Δ*XOC_2335* and Δ*XOC_2393* strains with plasmid-borne full-length genes restored swimming motility to wild type or near wild-type level (Fig. 1a). Since both XOC_2335 and XOC_2393 regulate swimming motility, a double mutant Δ*XOC_2335*/*XOC_2393* was constructed to investigate genetically the relationship between the two proteins in the control of swimming motility. As shown in Fig. 1b, the swimming ability of Δ*XOC_2335/XOC_2393* was similar to that of the mutant Δ*XOC_*2335 and was lower than that of Δ*XOC_2393* (Fig. 1b). The results demonstrated that *XOC_2335* and *XOC_2393* positively regulate the swimming motility of *Xoc*, with *XOC_2335* having the predominant effect.

### XOC_2102, XOC_2393 and XOC_4190 negatively regulate sliding motility

**Figure 2:**
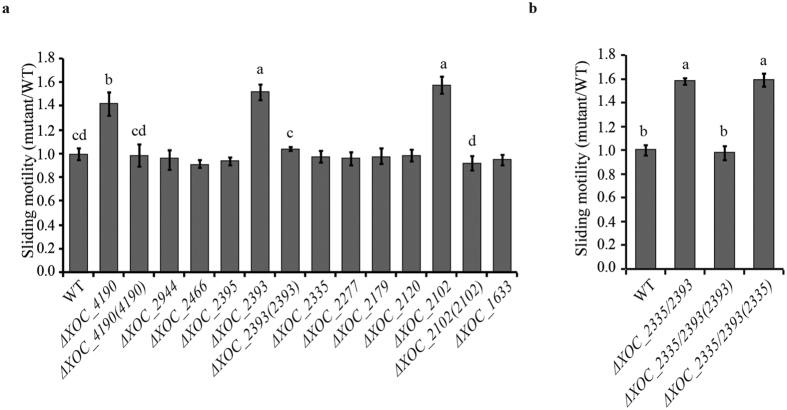
Effects of mutations of GGDEF-EAL protein-encoding genes on sliding motility of *Xoc*. The sliding motility of Δ*XOC_2102*, Δ*XOC_2393* and Δ*XOC_4190* was significantly increased compared with that of the wild-type strain. The complementation strains Δ*XOC_2102(2102)*, Δ*XOC_2393(2393)* and Δ*XOC_4190(4190)* restored sliding motility to the wild-type level. (**b**) The sliding motility of Δ*XOC_2335/XOC_2393* was significantly increased compared with the wild type and was restored by the full-length *XOC_2393* gene, but not by the full-length *XOC_2335* gene. The sliding motility of different strains was evaluated on SB medium plates with 0.6% noble agar after incubating at 28 °C for 4 d. The ratios of colony diameter of the mutant strains to the wild type were shown. Means ± SE are shown. The letters (a–d) indicate significant difference (*P* < 0.05) by Duncan’s multiple range test.

C-di-GMP signaling has been also demonstrated to be involved in control of type IV pili (T4P)-dependent motility in bacteria^12^. T4P is a thin filamentous structure present on outer surfaces of many bacteria. T4P has been shown to participate in twitching, sliding and several other important physiological processes such as adherence to surfaces^38^. The panel of mutants was tested for sliding motility on 0.6% agar SB medium plates. The Δ*XOC_2102*, Δ*XOC_2393* and Δ*XOC_4190* mutants had enhanced motility with colony diameters ~58%, ~52% and ~42% larger than the wild-type strain. Other mutants had no significant alteration in sliding motility. Complementation with the full-length *XOC_2102*, *XOC_2393* or *XOC_4190* gene restored the sliding motility of Δ*XOC_2102*, Δ*XOC_2393* and Δ*XOC_4190* to the wild-type level, respectively (Fig. 2a). Similarly, the Δ*XOC_2393*/*XOC_2335* mutant had a larger colony diameter on SB medium plates than the wild-type strain. The full-length *XOC_2393* gene, but not *XOC_2335*, restored the sliding motility of the double-gene deletion mutant (Fig. 2b). The results indicate that *XOC_2102*, *XOC_2393* and *XOC_4190* negatively regulate the sliding motility of *Xoc*.

### GGDEF-EAL domain proteins do not significantly affect EPS production, protease secretion and biofilm formation

Bacterial biofilm formation, EPS production and secretion of proteases are all important virulence factors in *Xanthomonas* spp.^39^,^40^. When the panel of single gene-deletion mutants was tested for these virulence-associated phenotypes, all strains produced similar amounts of EPS and biofilm biomass as the wild-type strain (see Supplementary Fig. S3). Similarly, the ability to synthesize and secrete proteases was not apparently altered in these mutants (see Supplementary Fig. S3). In addition, the double Δ*XOC_2335*/*XOC_2393* mutant exhibited similar phenotypes in EPS production, biofilm formation and protease secretion to the wild-type and single gene-deletion mutant strains (see Supplementary Fig. S3).

### XOC_2335, XOC_2393 and XOC_4190 are involved in regulating virulence to rice

**Figure 3:**
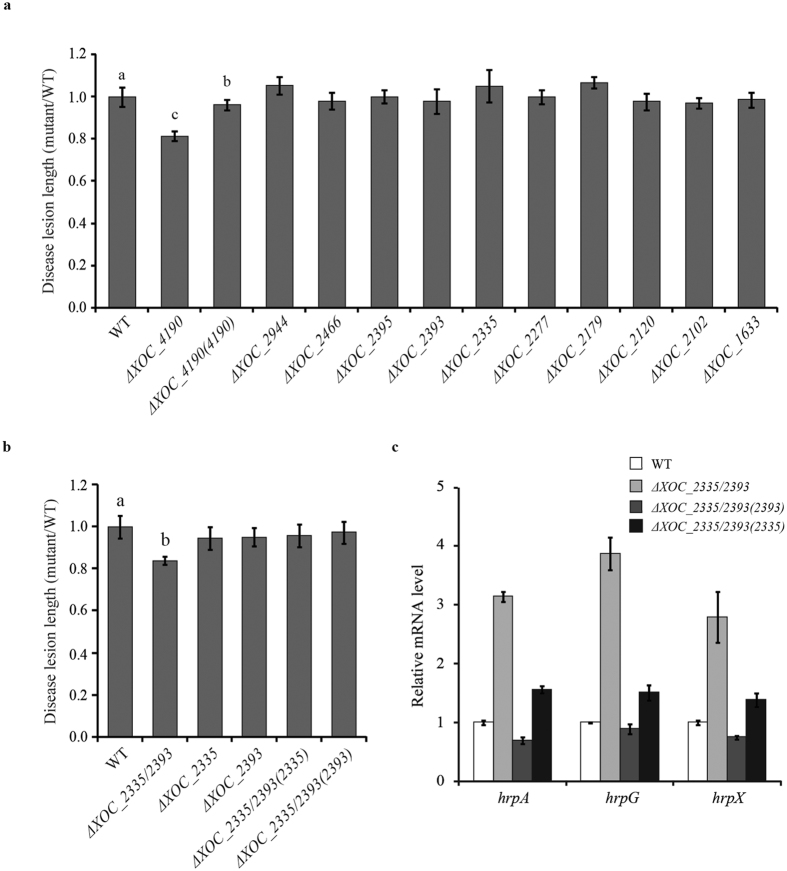
The effects of mutation of GGDEF-EAL protein-encoding genes on virulence of *X. oryzae* pv. *oryzicola* to rice. (**a**) The lesion length on the Δ*XOC_4190*-inoculated leaves of rice cv. Shanyou 63 was significantly shorter than that caused by the wild-type strain. Virulence of the Δ*XOC_4190* mutant was restored by the plasmid-borne full-length *XOC_4190* gene. Other single-gene deletion mutants have no altered virulence to rice compared with the wild type. (**b**) The lesion length caused by the Δ*XOC_2335/XOC_2393* double mutant was significantly shorter than that caused by the wild-type strain. Both XOC_2335 and XOC_2393 can restore virulence of the double-gene deletion mutant to the wild-type level. The length of disease lesions was measured at 14 d after pressure inoculation of the wild-type, mutant and complemented strains, respectively. The ratios of disease lesion length caused by the mutant strains to that caused by the wild-type strain were shown. Data are presented as means ± SE. The letters (a,b) indicate significant difference (*P* < 0.05) by Duncan’s multiple range test. (**c**) The effect of double-gene deletion of *XOC_2335* and *XOC_2393* on the expression of *hrp* genes in *Xoc*. Expression of *hrpA*, *hrpG* and *hrpX* in the wild-type (WT), Δ*XOC_2335*/*2393*, Δ*XOC_2335*/*2393(2393)*, Δ*XOC_2335*/*2393(2335)* strains was detected by qRT-PCR. 16S RNA was used as an internal control for data analyses. A significant increase of *hrpA*, *hrpG* and *hrpX* mRNA expression was detected in Δ*XOC_2335*/*2393* compared with the wild-type and complementation strains.

To investigate the roles of GGDEF-EAL proteins in *Xoc* virulence, the panel of mutants was pressure-inoculated into the leaves of rice plants. The length of disease lesions formed on the inoculated leaves was measured to evaluate bacterial virulence. The Δ*XOC_4190* mutant was the only single-gene deletion strain with altered virulence, causing disease lesions shorter than the wild type (Fig. 3a). The Δ*XOC_2335* and Δ*XOC_2393* strains, which did show alteration in swimming ability (see above), produced disease lesions of similar sizes to the wild type. These experiments were extended to test the virulence of the Δ*XOC_2335/XOC_2393* double mutant. The disease lesions caused by this strain were significantly shorter than those by the wild-type strain (Fig. 3b, see Supplementary Fig. S4). Complementation of the Δ*XOC_2335/XOC_2393* mutant with either *XOC_2335* or *XOC_2393* restored virulence of the mutant to the wild-type level (Fig. 3b, see Supplementary Fig. S4). Collectively, the results indicate that XOC_4190, XOC_2335 and XOC_2393 all contribute to virulence of *X. oryzae* pv. *oryzicola* to rice. Our previous study demonstrated that the *Xoc* Δ*rpfG* mutant had an altered expression of *hrp* regulon^31^. Therefore, we further determined the expression level of *hrpA*, *hrpG* and *hrpX* in the Δ*XOC_2335/XOC_2393* mutant using quantitative real-time reverse transcription PCR (qRT-PCR). The results showed that simultaneous deletion of *XOC_2335* and *XOC_2393* significantly increased the expression of *hrpA*, *hrpG* and *hrpX* under type III secretion-inducing conditions (Fig. 3c). Complementation of the double mutant by expression of *XOC_2335* and *XOC_2393 in trans* restored the expression of *hrpA*, *hrpG* and *hrpX* to the wild type or near to wild-type level (Fig. 3c). These results imply that XOC_2335 and XOC_2393 act synergistically and negatively control the expression of the type III secretion system (T3SS).

### Both GGDEF and EAL motifs of XOC_2335 are required for regulation of swimming motility

The phenotypic analyses indicate a role for XOC_4190, XOC_2335, XOC_2393 and XOC_2102 in virulence or motility of *Xoc*. As outlined above, proteins that contain tandem GGDEF and EAL domains can have DGC and/or PDE enzymatic activities. In some cases, both domains are enzymatically inactive but instead function as c-di-GMP effectors and/or are involved in protein-protein interactions. We used bioinformatic, genetic, biochemical and functional analyses (to include c-di-GMP binding) to reveal the possible mechanisms by which regulation by these different *Xoc* proteins may occur.

**Figure 4:**
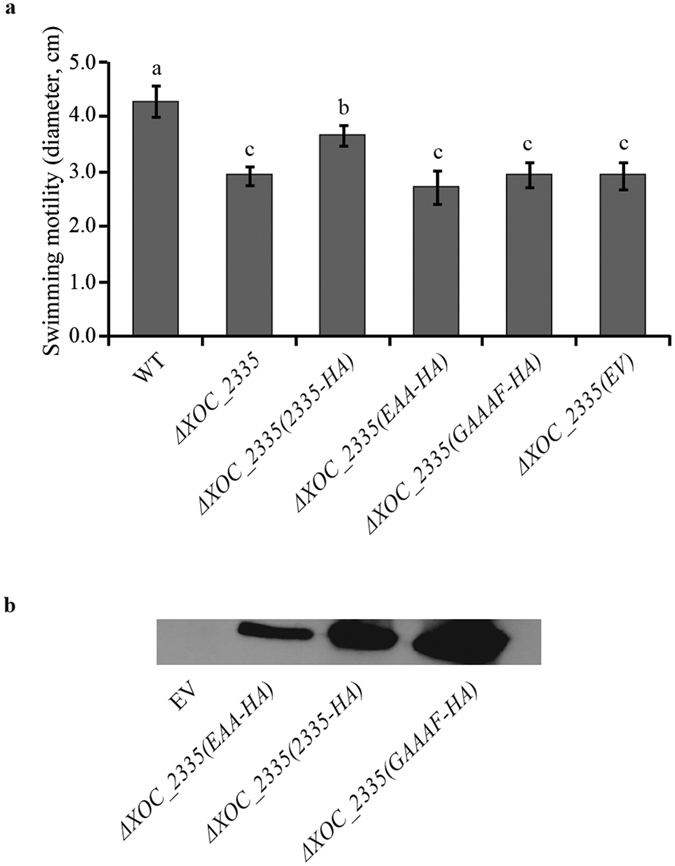
Point mutations in the GGDEF or EAL motifs of XOC_2335 abolish its function in the regulation of swimming motility. (**a**) The gene variant constructs with point mutations encoding XOC_2335^GADEF-GAAAF^-HA or XOC_2335^EAL-EAA^-HA variant proteins did not complement the swimming motility of Δ*XOC_2335* while the *XOC_2335-HA* construct partially restores the swimming motility of Δ*XOC_2335*. The colony diameters of different *Xoc* strains cultured on semisolid plates with 0.2% noble agar were shown. WT, the wild-type strain. (**b**) Expression of HA-tagged XOC_2335 and its variants XOC_2335^GADEF-GAAAF^ or XOC_2335^EAL-EAA^ in *Xoc* was detected by western blot analysis with an anti-HA antibody. EV indicates the wild-type strain transformed with the empty vector pVSP61 as negative control. The letters (a–c) indicate significant difference (*P* < 0.05) by Duncan’s multiple range test.

*In silico* analysis revealed that the GGDEF motif of XOC_2335 is degenerate, with the non-canonical GADEF motif at the active site. In contrast, all of the active site residues in the EAL domain^12^ were conserved, suggesting that overall XOC_2335 has a PDE activity. To determine the importance of the EAL motif for XOC_2335 function, a construct expressing a variant with EAA rather than EAL was made via site-directed mutagenesis. Complementation studies with this construct demonstrated that the XOC_2335^EAL-EAA^ variant was unable to restore swimming motility of Δ*XOC_2335* to the wild-type level (Fig. 4a). Another construct with the GADEF motif replaced by GAAAF in XOC_2335 was used to investigate the importance of the GGDEF motif. Similarly, Δ*XOC_2335* expressing the XOC_2335^GADEF-GAAAF^ variant had a similar swimming ability to the gene-deletion mutant (Fig. 4a). To confirm expression, constructs expressing variant proteins carrying HA tags were also made, allowing detection via immunoblotting. The results showed that the variant proteins with point mutations and the wild-type protein were all well expressed in *Xoc* (Fig. 4b). Furthermore, we demonstrated that expression of truncated XOC_2335-HA proteins, including XOC_2335ΔE without EAL domain or XOC_2335ΔG lacking GGDEF domain, in Δ*XOC_2335* did not restore swimming motility of the mutant (see Supplementary Fig. S5). Complementation analyses using the XOC_2335 variants demonstrated that both non-canonical GADEF and conserved EAL motifs are essential for XOC_2335 function *in vivo*. Unfortunately, multiple attempts to express and purify XOC_2335 in *E. coli* failed because the protein was insoluble, precluding further studies.

### The GGDEF and EAL domains of XOC_ 2102 and XOC_4190 are degenerate and enzymatically inactive

**Figure 5:**
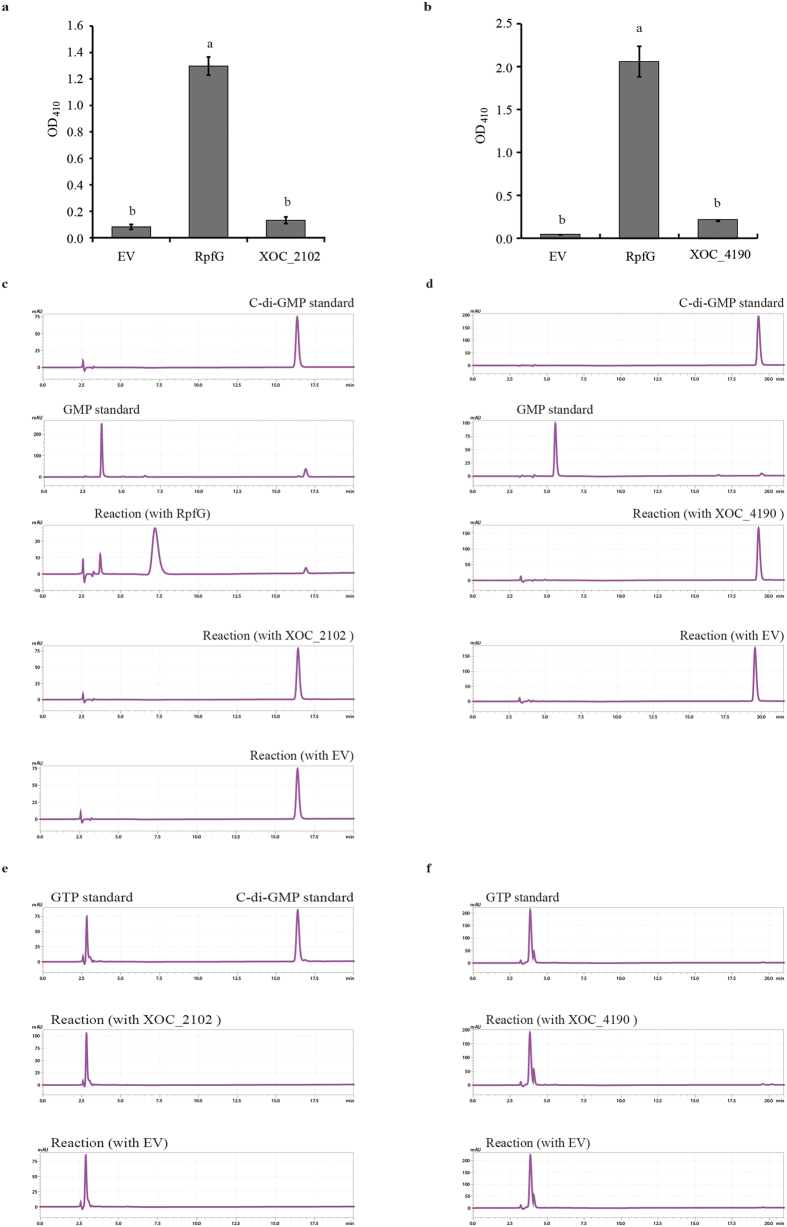
XOC_2102 and XOC_4190 are phosphodiesterase- and diguanylate cyclase-inactive. The PDE activity of the purified XOC_2102 (**a**) and XOC_4190 (**b**) was detected by colorimetric assays. No yellow degradation product *p*-nitrophenol was detected when the purified XOC_2102 (**a**) or XOC_4190 (**b**) was incubated with the colorless phosphodiesterase substrate bis(*p*-nitrophenyl) phosphate. As a positive control, the known PDE RpfG degraded the substrate into the yellow product that was detected spectrophotometrically at 410 nm. The PDE activity of the purified XOC_2102 (**c**) and XOC_4190 (**d**) was detected by HPLC assays. No degraded product was detected when XOC_2102 and XOC_4190 were incubated with c-di-GMP. In the same reaction buffer, two hydrolytic products pGpG and GMP were detected after RpfG was incubated with c-di-GMP for 6 h (**c**). The DGC activity of XOC_2102 (**e**) and XOC_4190 (**f**) was assayed by HPLC. No synthetic c-di-GMP was detected when the purified XOC_2102 (**e**) and XOC_4190 (**f**) were incubated with GTP in the assay buffer for 6 h. GTP and c-di-GMP were loaded and detected as standards. The eluant from empty vector (EV)-transformed *E. coli* cells through nickel column was used as a negative control.

Bioinformatic analysis indicates that the GGDEF and EAL domains of both XOC_2102 and XOC_4190 are degenerate, with the variant motifs NDNST and QVL respectively in XOC_4190 and GEHSF and QAF respectively in XOC_2102. To experimentally verify these predictions, XOC_2102 and XOC_4190 with N-terminal His6-tags were expressed in *E. coli* and the recombinant proteins were purified as described in Materials and Methods. The purified proteins were first tested for the PDE activity through colorimetric assay. In this assay, the PDE activity was evaluated by the hydrolysis of the colorless substrate, bis(*p*-nitrophenyl) phosphate, into yellow *p*-nitrophenol that can be detected spectrophotometrically at 410 nm. Incubation of His6-XOC_2102 or His6-XOC_4190 with bis(*p*-nitrophenyl) phosphate did not give production of *p*-nitrophenol that was not significantly different from the mock control (no enzyme) (Fig. 5a,b). As a positive control, RpfG efficiently converted bis(*p*-nitrophenyl) phosphate into *p*-nitrophenol (Fig. 5a,b). The DGC or PDE activity of His6-XOC_2102 and His6-XOC_4190 was also tested by reverse phase high-performance liquid chromatography (HPLC) separation of reaction mixtures in which the purified proteins were incubated with GTP or c-di-GMP. No synthesis or degradation of c-di-GMP was detected under our experimental conditions (Fig. 5c–f). By contrast, a significant amount of the degraded product pGpG was produced when RpfG was incubated with c-di-GMP (Fig. 5c)^31^. Taken together, these results indicate that XOC_2102 and XOC_4190 with degenerate GGDEF and EAL domains are enzymatically inactive.

### XOC_2102 and XOC_4190 bind to c-di-GMP *in vitro*

**Figure 6:**
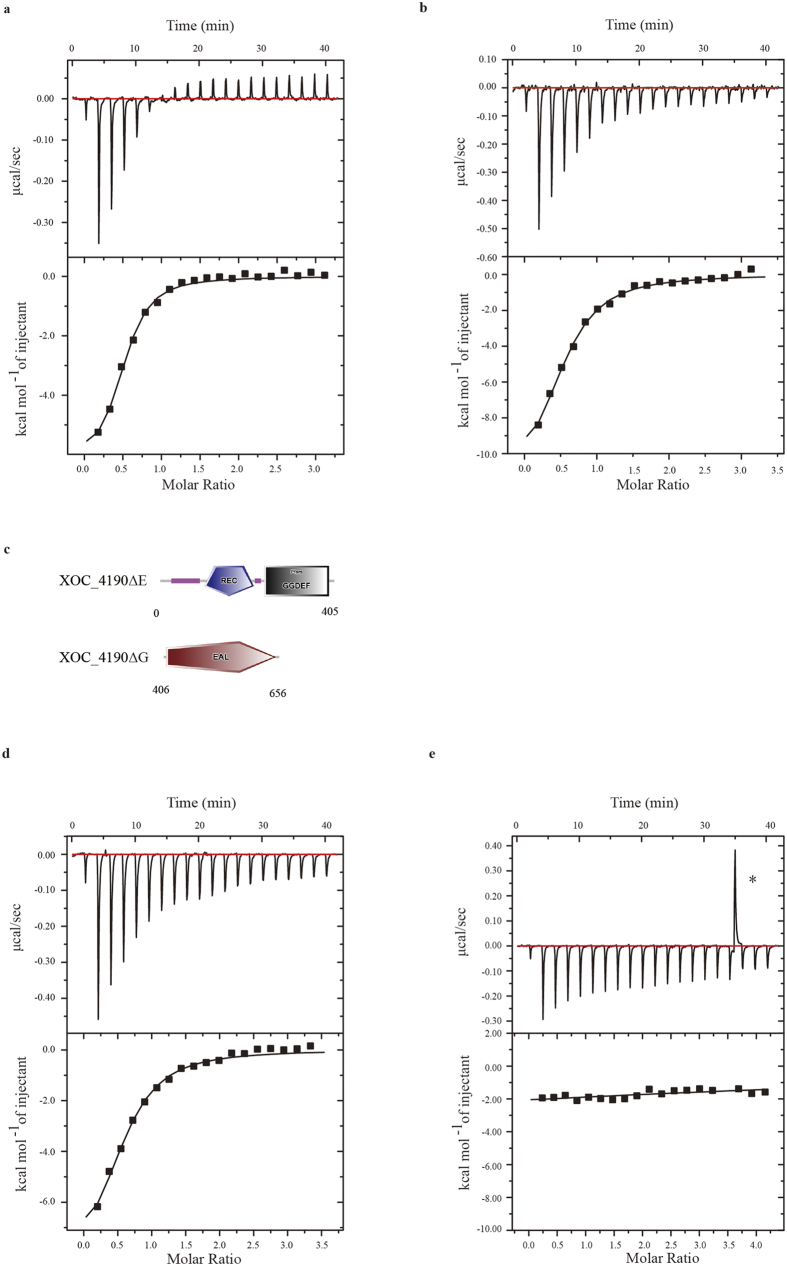
XOC_2102 and XOC_4190 bind to c-di-GMP *in vitro*. Isothermal titration calorimetry (ITC) measurement for the interaction of c-di-GMP and XOC_2102 (**a**) or XOC_4190 (**b**). The data indicate that the dissociation constants (*K*_d_) are 2.90 ± 0.13 μM or 4.59 ± 0.36 μM, respectively. (**c**) Schematic representation of the two truncated XOC_4190 proteins. XOC_4190ΔE, the truncated XOC_4190 without EAL domain; XOC_4190ΔG, the truncated XOC_4190 with the only EAL domain. ITC measurement for the interaction between c-di-GMP and XOC_4190ΔG (**d**) or XOC_4190ΔE (**e**). The data indicate that the *K*_d_ is 4.46 ± 0.28 μM for interaction between c-di-GMP and XOC_4190ΔG. Top panels, the titration calorimetry of the proteins with c-di-GMP at room temperature; lower panels, normalized ITC data for titrations versus molar ratio of c-di-GMP and the proteins.

As pointed out above, proteins containing degenerate GGDEF and EAL domains can serve as c-di-GMP receptors^19^,^41^. Isothermal titration calorimetry (ITC) was performed to assess the binding of c-di-GMP to XOC_2102 and XOC_4190. In this assay, the purified recombinant His6-XOC_2102 and His6-XOC_4190 were titrated with c-di-GMP at room temperature. The dissociation constants (*K*_d_) were determined after analysis of the normalized ITC curve by the Origin software^42^. The data indicate that XOC_2102 binds to c-di-GMP with a *K*_d_ of 2.90 ± 0.13 μM, and that XOC_4190 binds to c-di-GMP with a *K*_d_ of 4.59 ± 0.36 μM (Fig. 6a,b).

To further investigate c-di-GMP binding to XOC_4190, two truncated proteins, XOC_4190∆G lacking the GGDEF domain and XOC_4190∆E without the EAL domain, were expressed and purified (Fig. 6c). Both truncated proteins were investigated for c-di-GMP binding via ITC assays. It was shown that XOC_4190∆G still bound to c-di-GMP with a *K*_d_ of 4.46 ± 0.28 μM, while XOC_4190∆E completely lost the c-di-GMP binding ability (Fig. 6d,e). The results indicate that the EAL domain, but not the GGDEF domain is required for binding of c-di-GMP by XOC_4190.

### *Xoc* Δ*XOC_2335*, Δ*XOC_2393* and Δ*XOC_2335/XOC_2393* mutants, but not Δ*XOC_4190*, have elevated intracellular c-di-GMP levels

**Figure 7:**
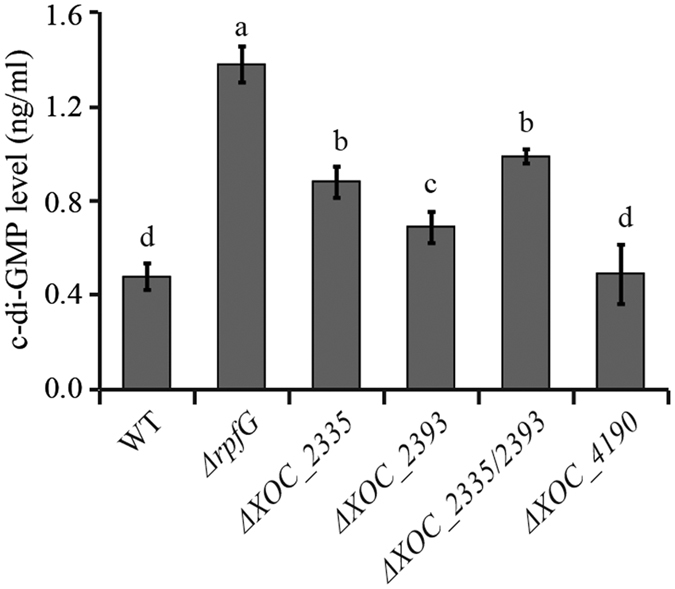
Measurement of intracellular c-di-GMP levels in wild-type or mutant *Xoc* strains. Assays were performed as described in the Methods. The c-di-GMP concentrations were quantified in the wild type (WT), Δ*rpfG*, Δ*XOC_2335*, Δ*XOC_2393*, Δ*XOC_2335*/*2393* and Δ*XOC_4190* mutants. An increased c-di-GMP concentration was revealed in Δ*rpfG*, Δ*XOC_2335*/*2393*, Δ*XOC_2335*, and Δ*XOC_2393* in comparison with the wild-type strain. The c-di-GMP level in Δ*XOC_4190* was similar to that in the wild-type strain. Data are presented as means ± SE. The letters (a–d) indicate significant difference (P < 0.05) by Duncan’s multiple range test.

To determine if mutation of these GGDEF-EAL proteins affects the intracellular level of c-di-GMP, we quantified the c-di-GMP concentration in the wild type and multiple gene-deletion mutants using liquid chromatography-mass spectrometry. As shown in Fig. 7, Δ*rpfG* has the highest c-di-GMP concentration among the wild-type and tested mutant strains, consistent with previous findings that RpfG functions as a PDE and the Δ*rpfG* mutant showed more drastic changes phenotypically^28^,^31^. Our results also showed an elevated c-di-GMP concentration in Δ*XOC_2335*, Δ*XOC_2393* and Δ*XOC_2335/XOC_2393* mutants compared with the wild-type strain. Meanwhile, the double mutant had a higher level of c-di-GMP than the single gene deletion mutants (Fig. 7). By contrast, no change in the c-di-GMP level was detected in the Δ*XOC_4190* mutant, consistent with the results from PDE and DGC activity assays. These results suggest that XOC_2335 and XOC_2393, but not XOC_4190, function to degrade c-di-GMP and are indeed PDEs.

## Discussion

C-di-GMP is an important secondary messenger in phytopathogenic bacteria that has pleiotropic effects on virulence-associated cellular processes^3^. In the present study, we have addressed one facet of c-di-GMP regulation in *Xoc* by assessing the contribution of eleven tandem GGDEF-EAL proteins to bacterial virulence and virulence-associated traits. Three of these proteins (XOC_4190, XOC_2335 and XOC_2393) were implicated in the regulation of *Xoc* virulence and together with XOC_2102 were shown to control bacterial swimming and sliding motilities, but did not significantly affect other virulence-related functions such as biofilm formation, EPS production and protease secretion. The action of these four proteins in regulation appears to be different. XOC_2102 and XOC_4190 do not have detectable DGC or PDE activity but bind to cyclic di-GMP with high affinity, suggesting that they may act as effector proteins for the nucleotide. In contrast, XOC_2335 and XOC_2393 may be PDEs that can influence intracellular levels of c-di-GMP.

Virulence was assessed following direct inoculation of bacteria into rice leaves. This assay bypasses the earlier phases of the disease cycle where bacteria have an epiphytic lifestyle before entry into the host extracellular spaces through stomata and wounds. It may well be that other GGDEF-EAL domain proteins control factors that are important in this early phase of disease. In this context it should be noted that many GGDEF-EAL proteins carry other sensory and signal transduction domains and may regulate virulence-associated traits in response to environmental cues only found in naturally infected plants. The absence of an effect of mutation on known virulence factors when assayed *in vitro* may not reflect what occurs *in planta* during disease. For example, the PAS-GGDEF-EAL domain protein XC2324 of *X. campestris* pv. *campestris* may sense molecular oxygen through binding to the PAS domain, only causes a significant reduction in the synthesis of the virulence factors endoglucanase and endomannanase under low oxygen condition^27^.

Quantification of the intracellular c-di-GMP concentration in Δ*XOC_2335*, Δ*XOC_2393* and Δ*XOC_2393/XOC_2335* mutants showed that these mutants all had higher c-di-GMP levels than the wild type (Fig. 7), indicating that XOC_2335 and XOC_2393 are indeed PDEs. Interestingly, the double Δ*XOC_2393/XOC_2335* mutant was attenuated in virulence to rice, although Δ*XOC_2393* and Δ*XOC_2335* exhibited no significant alteration in virulence. These results suggest that XOC_2335 and XOC_2393 might function redundantly or additively, consistent with the finding that the double mutant has a higher c-di-GMP level than the single gene mutants. In a previous study, we demonstrated that deletion of *rpfG* abolished *Xoc* virulence to rice and RpfG negatively regulated the expression of T3SS^31^. Consistently, *hrp* operon was shown to be significantly up-regulated in the Δ*XOC_2393/XOC_2335* mutant (Fig. 3c). Together with the finding that the c-di-GMP level is dramatically elevated in the Δ*rpfG* mutant, these results suggest that higher concentration of c-di-GMP is involved in positive regulation of T3SS expression in *Xoc*. In contrast, PdeR, a homolog of XOC_2393, with PDE activity, was required for the expression of the T3SS that is essential for bacterial virulence in *Xoo*^19^. It warrants to be further investigated why these PDEs might regulate T3SS expression in different ways in *Xoo* and *Xoc*.

The GGDEF-EAL domain proteins affecting virulence in *Xoc* have homologs in *Xoo*, but the effects of mutation of the encoding genes are different. As noted above, XOC_2393 is a homolog of PdeR in *Xoo* PXO99^A^. The *pdeR* mutant is attenuated in virulence to rice and produces much less exopolysaccharide than the wild type^19^. By contrast, under our experimental conditions the Δ*XOC_2393* mutant exhibited no significant difference from the wild type in phenotypes other than motility. XOC_4190 and XOC_2102 are homologous to PXO_02944 and Filp of *Xoo*, respectively. In contrast to Δ*XOC_4190* that has attenuated virulence to rice, Δ*PXO_02944* exhibits increased EPS production, biofilm formation and also elevated virulence to rice^43^. Δ*XOC_2102* and Δ*filp* strains showed similar phenotypes, with altered motility, but no effects on EPS production or biofilm formation. However, the *filp* mutant is attenuated in virulence to rice unlike the Δ*XOC_2102* strain. Different phenotypes caused by mutation of homologous genes in xanthomonads have been reported previously^27^,^44^. Several reports suggest that DSF signaling regulates virulence-associated traits in a completely different pattern in *Xoo* and *Xcc*^45^,^46^,^47^. The difference might be due to genetic divergence and different infection styles among the *Xanthomonas* species.

Bacteria exhibit several types of motilities, such as swimming, twitching and sliding motilities under various conditions^12^. A body of work has implicated GGDEF-EAL domain proteins in the regulation of these different modes of motility. For example, mutation of XC2161 in *X. campestris* pv. *campestris* reduces pilus-dependent motility, while loss of another GGDEF-EAL protein XC2226 causes an opposite phenotype^27^. FimX of *P. aeruginosa* is reported to be involved in type IV pilus-based motility^12^. In general, higher levels of c-di-GMP suppresses swimming motility^12^. Our finding that deletion of the genes encoding the putative PDEs XOC_2335 and XOC_2393 caused the elevated intracellular c-di-GMP level and reduced swimming motility is consistent with this contention. Bioinformatic analysis of XOC_2393 indicates that it has a degenerate GGDEF domain, with a GSDEM motif, but that key residues in the EAL domain required for active PDEs are all conserved. XOC_2393 shares 95.2% amino acid sequence similarity to PdeR in *X. oryzae* pv. *oryzae* PXO99^A^ and the EAL domains of the two proteins are identical. Intriguingly, PdeR has been shown to have PDE activity *in vitro*^44^. Complementation experiments with point mutations in GGDEF or EAL motifs demonstrated that both motifs were required for XOC_2335 function in regulating swimming motility. In *C. crescentus*, the GGDEF-EAL protein CC3396 is able to bind GTP through degenerate GGDEF domain and then activates the PDE activity in the neighboring EAL domain^18^. Therefore, it is interesting to further investigate whether XOC_2335 and XOC_2393 function in swimming motility with a similar mechanism to CC3396. Unfortunately, multiple attempts to express and purify XOC_2335 and XOC_2393 in *E. coli* failed because the proteins were insoluble.

Interestingly, we demonstrated that XOC_2335 positively controlled swimming ability, but did not affect sliding motility. By contrast, XOC_2393 regulated both swimming and sliding motilities in the opposite way. Therefore, the effect of XOC_2335 and XOC_2393 on phenotypes was not completely redundant. Swimming motility is mediated by flagella, while sliding motility is mediated by the type IV pili. Speculatively, XOC_2393 might regulate the pili-mediated sliding motility in a c-di-GMP-independent manner. These findings indicate a complexity in regulation that might reflect an emerging concept of the multi-functionality of c-di-GMP signaling proteins, which can have a regulatory action exerted through protein-protein interactions that is independent of their enzymatic activity in cyclic di-GMP turnover.

Bioinformatic analysis indicates that XOC_2102 and XOC_4190 with degenerate GGDEF and EAL domains might not function as a DGC or PDE, which is consistent with the DGC and PDE enzymatic assays (Fig. 5). The hypothesis is also supported by the fact that the c-di-GMP level is not altered in Δ*XOC_4190* mutant (Fig. 7). Previous studies showed that some enzymatically inactive GGDEF-EAL domain proteins, such as FimX from *P. aeruginosa*, LapD from *P. fluorescens*, and Filp from *X. oryzae* pv. *oryzae* PXO99^A^ can act as a major class of c-di-GMP receptors in different bacteria. These proteins bind to c-di-GMP with high-affinity through the degenerate and enzymatically inactive C-terminal EAL domains and serve as the receptors of this signal molecule^19^,^21^,^37^. In this study, ITC assays clearly showed that XOC_4190 and XOC_2102 bound to c-di-GMP. Furthermore, we further revealed that the only EAL domain of XOC_4190 can bind to c-di-GMP, while the GGDEF domain cannot. Therefore, several of these *Xoc* proteins with degenerate GGDEF-EAL domains might function in c-di-GMP signaling through serving as c-di-GMP effectors.

In this study, we systemically analyzed the functions of GGDEF-EAL proteins in *Xoc*. XOC_4190 was shown to be an essential regulator of bacterial virulence likely through functioning as a c-di-GMP receptor. Both XOC_2335 and XOC_2393 individually regulate bacterial motility and together control *Xoc* virulence. These novel findings suggest new questions for further research. How do c-di-GMP signaling proteins regulate various types of bacterial motility in opposite ways? How is c-di-GMP signaling involved in virulence regulation? Identification of protein-protein interactions involved in signaling and determination of the crystal structure of the putative effectors in complex with c-di-GMP will help to elucidate the molecular mechanisms underlying the diverse regulatory functions of GGDEF-EAL proteins.

## Methods

### Bacterial strains and culture conditions

The *X. oryzae* pv. *oryzicola* strain RS105 and its mutants were cultured in nutrient broth (NB) medium (3 g/L beef extract, 1 g/L yeast extract, 5 g/L tryptone, 10 g/L sucrose) at 28 °C. Yeast cultures were grown in YPDA medium (20 g/L Bacto^TM^ Peptone, 10 g/L yeast extract, 20 g/L glucose, 30 mg/L adenine) at 28 °C. Antibiotics were used at the following concentrations: ampicillin, 100 μg/ml; kanamycin, 50 μg/ml; rifampin, 25 μg/ml. All gene constructs were confirmed by sequencing.

### Mutant construction and complementation of *Xoc* mutant strains

Non-marker homologous recombination was used to construct gene-deletion mutants of *Xoc* as described previously^31^. Briefly, ~1 kb long flanking regions upstream and downstream of open reading frames (ORFs) of the target genes were amplified by PCR with specifically designed primers (see Supplementary Table S2). PCR products were gel purified and added together into a fusion PCR. The resultant PCR fragments were cloned into pUFR80, which carries the *sacB* suicide gene^48^. The constructed plasmids were then conjugated into *Xoc* RS105 through triparental mating. The recombinant conjugants resulted from double cross-over events were screened on nutrient agar (NB medium with 1.5% agar) plates with 5% sucrose. The gene-deletion genotypes of sucrose-insensitive *Xoc* colonies were identified through colony PCR and were then subjected to confirmation via Southern blot analyses.

To construct complementation strains of *Xoc* mutants, full-length genes including native promoters were amplified by PCR using designed primers (see Supplementary Table S2). The amplified products were cloned into the wide host-range vector pVSP61^49^. After being confirmed by sequencing, the constructs were individually conjugated into the corresponding mutants and successful conjugants were then selected on kanamycin-containing NA plates.

### Southern blot analysis

Southern blot analysis was performed according to standard protocols in molecular biology^50^. Briefly, genomic DNA was isolated from the wild-type and mutant strains of *Xoc* using a genomic DNA isolation kit (New Industry Company, Beijing, China) and was then digested with appropriate restriction enzymes overnight. After separated by agarose gel, digested genomic DNA was transferred onto Hybond-N nylon membrane (GE Healthcare) and was then hybridized with the ^32^P-labeled gene fragments that were amplified by PCR using the primers listed in the Supplementary Table S2.

### Biofilm quantification

Biofilm masses of *Xoc* cultures were quantified as described previously^31^. Briefly, bacterial strains were cultured in 5 ml L medium (10 g/L tryptone, 5 g/L yeast extract, 5 g/L NaCl and 1 g/L glucose) in the borosilicate glass tubes. After 7 d of incubation at quiescent state at 28 °C, cultured cells were stained with 10% crystal violet (CV) for 15 min. The tubes were rinsed with H_2_O carefully to remove the surplus dye. The CV-stained biofilm attached to the tubes was solubilized in 90% ethanol and quantified by spectrophotometry at 590 nm.

### Exopolysaccharide production

Exopolysaccharide (EPS) production in *Xoc* cultures was determined as described previously^31^,^51^. Overnight cultures of *Xoc* were collected and re-suspended in sterile water to an OD600 of ~1.0. The cells were diluted at 1:1, 000 with M210 medium (8 g/L casein enzymatic hydrolysates, 4 g/L yeast extract, 5 g/L sucrose, 3 g/L KH_2_PO_4_, 0.3 g/L MgSO_4_·7H_2_O) and cultured further for 30 h. The supernatants (10 ml) were collected after centrifugation at 12,000 rpm for 5 min. EPS was precipitated by mixing the supernatant with two volumes of absolute ethanol and incubating at −20 °C overnight. The precipitate was collected by centrifugation at 10,000 rpm for 5 min and fully dried at 55 °C before weighing.

### Protease secretion assay

The secretion of extracellular proteases of *Xoc* strains was assayed as described elsewhere^52^. Briefly, bacteria were cultured on the water agar plates containing 1% (w/v) skimmed milk at 28 °C for 4 d. The diameter of clearing zones around the colonies formed by the proteolytic degradation of skimmed milk was measured to assess the activity of secreted proteases.

### Bacterial motility assays

The swimming motility of *Xoc* strains was determined on semisolid medium plates containing 0.2% noble agar, 0.03% yeast extract and 0.03% bacto peptone^53^. *Xoc* strains were inoculated into the center of the plates by pipetting. After culturing at 28 °C for 4 d, the diameter of bacterial colonies was measured. The sliding motility of *Xoc* was evaluated by the diameter of bacterial colonies after *Xoc* strains were incubated on SB medium plates (5 g/L bacto peptone, 5 g/L yeast extract, 1 g/L L-glutamic acid, and 0.6% noble agar) for 4 d at 28 °C^38^.

### Virulence assay of *Xoc* to rice

Virulence assays were performed as described previously^25^. Briefly, overnight grown cells were collected and re-suspended in sterile distilled water to cell density of OD600 = 0.3. Bacterial suspensions were pressure-infiltrated into the leaves of 6-week-old rice plants using needleless syringes. The length of disease lesions was measured at 14 d after inoculation. At least 10 inoculated leaves were measured for each of the tested *Xoc* strains.

### RNA isolation and quantitative real-time RT-PCR

Overnight *Xoc* cultures were diluted in the *hrp*-inducing XOM3 medium^54^ to an OD_600_ of 0.08 and grew further till OD_600_ = 0.6. The cells were then harvested to isolate RNA using TransZol Up Plus RNA Kit (Transgen, Beijing, China) following the manufacturer’s instructions. Quantitative real-time RT-PCR (qRT-PCR) was performed as described previously with minor modifications^31^. Bestar^®^ SYBRGreen qPCR Mastermix (DBI^®^ Bioscience, Shanghai, China) was used here in qRT-PCR reactions to quantify the transcript levels. Expression of 16S rRNA was used as the internal reference for data analysis.

### Site-directed mutagenesis and gene constructs encoding truncated proteins

Site-directed mutagenesis was performed through circular PCR following the manufacturer’s instructions (Stratagene). Briefly, PCR was performed with *Pfu* Ultra DNA polymerase using the pUC19-*XOC_2335* plasmid DNA as template. After the methylated template DNA was digested with *Dpn* I for 3 h at 37 °C, PCR products were transformed into *E. coli* DH5α cells. The plasmid DNA with point mutations were isolated and subject to confirmation by sequencing. The mutated gene fragments were released from the pUC19 constructs with *Bam*H I and *Sac* I and were then re-ligated into pVSP61. The native promoter and coding sequences for GGDEF domain of XOC_2335 were amplified with the primer set 2335-*Sac* I-HA-F/2335-∆E-*Hin*d III-HA-R (see Supplementary Table S2). The promoter and coding sequences for EAL domain of XOC_2335 were amplified with the primer sets 2335-*Sac* I-HA-F/2335-∆G-R and 2335-∆G-F/2335-*Hin*dIII-HA-R, respectively (see Supplementary Table S2). Two fragments were fused by overlap extension PCR and the resultant PCR products were cloned into pVSP61^49^.

### Protein expression and purification

The ORFs of *XOC_4190* and *XOC_2102* were amplified from *Xoc* RS105 genome by PCR using primer sets listed in the Supplementary Table S2. The amplified fragments were inserted into pQE30 (Qiagen) after the digestion with *Bam*H I and *Hin*d III. The truncated coding sequences for GGDEF and EAL domains of XOC_4190 (1–405 and 406–656 amino acid residues, respectively) were amplified with the primer sets XOC_4190∆E-F/XOC_4190∆E-R and XOC_4190∆G-F/XOC_4190∆G-R, respectively (see Supplementary Table S2). The PCR fragments were sub-cloned into the expression vector pET28a (Novagen, Madison, WI) after gel purification and digestion. The pQE30 and pET28a constructs were transformed into the *E. coli* strains XL1-blue and BL21(DE3), respectively. To induce expression of the target proteins, isopropyl β-D-thiogalactopyranoside (IPTG, 0.1 mM) was added into cell cultures when OD600 reached 0.5 ~ 0.7. After 4 ~ 5 h of induction at room temperature, cultured cells were collected by centrifugation. For protein purification, cell pellets were re-suspended in lysis buffer (20 mM Tris-Cl, 50 mM NaCl, and 10 mM imidazole, pH 8.0) and then subject to sonication. The supernatant was prepared by centrifugation at 10,000 g for 20 min and loaded onto nickel-nitrilotriacetic acid agarose superflow columns (Qiagen), which were thoroughly rinsed with washing buffer (20 mM Tris-Cl, 50 mM NaCl, and 20 mM imidazole, pH 8.0). The bound His6-tagged protein was eluted with elution buffer (20 mM Tris-Cl, 50 mM NaCl, and 250 mM imidazole, pH 8.0) and dialyzed extensively in 10 mM Tris-Cl (pH 8.0). The concentration of purified proteins was determined using Pierce^TM^ BCA protein assay kit (Thermo Scientific, IL, USA).

### Immunoblotting

Total protein extracts were separated by a 12% polyacrylamide gel after boiling for 10 min. The proteins were electrophoretically transferred onto Immobilon-P membrane (Millipore) for immunoblotting with a horseradish peroxidase-conjugated anti-HA antibody (Roche). The membranes were then incubated with the eECL Western Blot chemiluminescent substrate (CWBio, China) for 5 min, and then exposed to X-ray films.

### Phosphodiesterase colorimetric assay

*In vitro* purified proteins were assayed for the PDE activity using colorimetric assays^55^. Briefly, purified proteins (20 μg) were incubated with the PDE substrate, 5 mM bis(*p*-nitrophenyl) phosphate, at 37 °C for 1.5 h in assay buffer (50 mM Tris-Cl, 1 mM MnCl_2_, pH 8.5). The yellow product was quantified using spectrophotometer at OD_410_.

### Enzymatic assays by high-performance liquid chromatography

The DGC and PDE activities were also assayed using high-performance liquid chromatography (HPLC) as described^31^,^55^. The enzyme activities were investigated using 20 μg of purified proteins in a buffer containing 25 mM Tris-Cl, pH 7.9, 250 mM NaCl, and 10 mM MgCl_2_. The PDE activity was tested using 100 μM of c-di-GMP as substrates, while the DGC activity was determined by replacing c-di-GMP with GTP (100 μM). The reaction mixture was incubated at 37 °C for 6 h and then stopped by boiling for 3 min. After centrifugation at 15,000 g for 2 min, the supernatant was filtered through a 0.22 μm membrane. Each sample (20 μl) was injected into a reverse phase C18 column (250 × 4.60 mm, 5 μm; Phenomenex, USA) with a Shimadzu 10AT system (Shimadzu Co., Ltd, Japan). The material was eluted at a flow rate of 1 ml/min with a 1%/min linear gradient of 0 ~ 20% methanol in 20 mM potassium phosphate buffer (pH 5.8). The products were visualized under 254-nm UV light.

### Isothermal titration calorimetry assay

Isothermal titration calorimetry (ITC) assays were performed on a MicroCal iTC200 colorimeter (MicroCal, Northampton, MA) to investigate the binding of c-di-GMP to *in vitro* purified proteins^42^. Titration calorimetry was performed at 25 °C in the assay buffer containing 20 mM Tris-Cl (pH 8.0), 500 mM NaCl and 250 mM imidazole. In brief, c-di-GMP solution (100 ~ 500μM, 2 μl aliquots) was injected at 2 min intervals via a 40 μl syringe into the sample cell containing the purified proteins (10 ~ 50 μM). ITC data were analyzed by integrating heat effects after being normalized to the amount of injected c-di-GMP. Curve fitting was performed based on a single-site binding model to determine the dissociation constants (*K*_d_) using the MicroCal ORIGIN version 7.0 software.

### Determination of intracellular c-di-GMP concentration

Intracellular c-di-GMP levels in the wild-type and mutant strains were determined by liquid chromatography tandem mass spectrometry (LC-MS/MS) as described previously^56^. Briefly, overnight bacterial cultures were diluted to 1:100 into 90 ml LB media in a flask and grew further till OD_600_ ~ 0.8. The cultures were collected by centrifugation and were then re-suspended with 100 μl of extraction buffer (40% methanol and 40% acetonitrile in 0.1 N formic acid) per 48 mg of wet cell weight. The slurries were incubated 30 min at −20 °C and insoluble material was removed by centrifugation at 4 °C. The supernatants were neutralized by the addition of 4 μl of 15% NH_4_ HCO_3_ per 100 μl of sample. Each sample (100 μl) was analyzed using LC-MS/MS.

### Statistical Analysis

Significant differences in various phenotypes among different strains (*P* < 0.05) were determined by Duncan’s multiple range test using SAS program.

## Additional Information

**How to cite this article**: Wei, C. *et al*. A systematic analysis of the role of GGDEF-EAL domain proteins in virulence and motility in *Xanthomonas oryzae* pv. *oryzicola*. *Sci. Rep*. **6**, 23769; doi: 10.1038/srep23769 (2016).

## Supplementary Material

Supplementary Information
