# Supplementary material for: A systematic analysis of the role of GGDEF-EAL domain proteins in virulence and motility in Xanthomonas oryzae pv. oryzicola
Source: Sci Rep. 2016 Apr 7;6:23769. doi: 10.1038/srep23769 (PMC4823724; doi:10.1038/srep23769)
Supplement: Supplementary Information [file srep23769-s1.pdf]

A systematic analysis of the role of GGDEF-EAL domain proteins in virulence and motility in *Xanthomonas oryzae* pv. *oryzicola*

Chao Wei[1], Wendi Jiang[1], Mengran Zhao[1], Junjie Ling[1], Xin Zeng[1], Jun Deng[1], Dongli Jin[1], John Maxwell Dow[2], Wenxian Sun[1,*]

[1]Department of Plant Pathology and the Ministry of Agriculture Key Laboratory for Plant Pathology, China Agricultural University, Beijing, China

[2]School of Microbiology, BioSciences Institute, University College Cork, Cork, Ireland

*Corresponding author: Wenxian Sun

Department of Plant Pathology China Agricultural University

2 West Yuanmingyuan Rd., Haidian District

Beijing 100193, China

Telephone: +86 10 6273 3532;

Fax: +86 10 6273 3532;

E-mail: wxs@cau.edu.cn

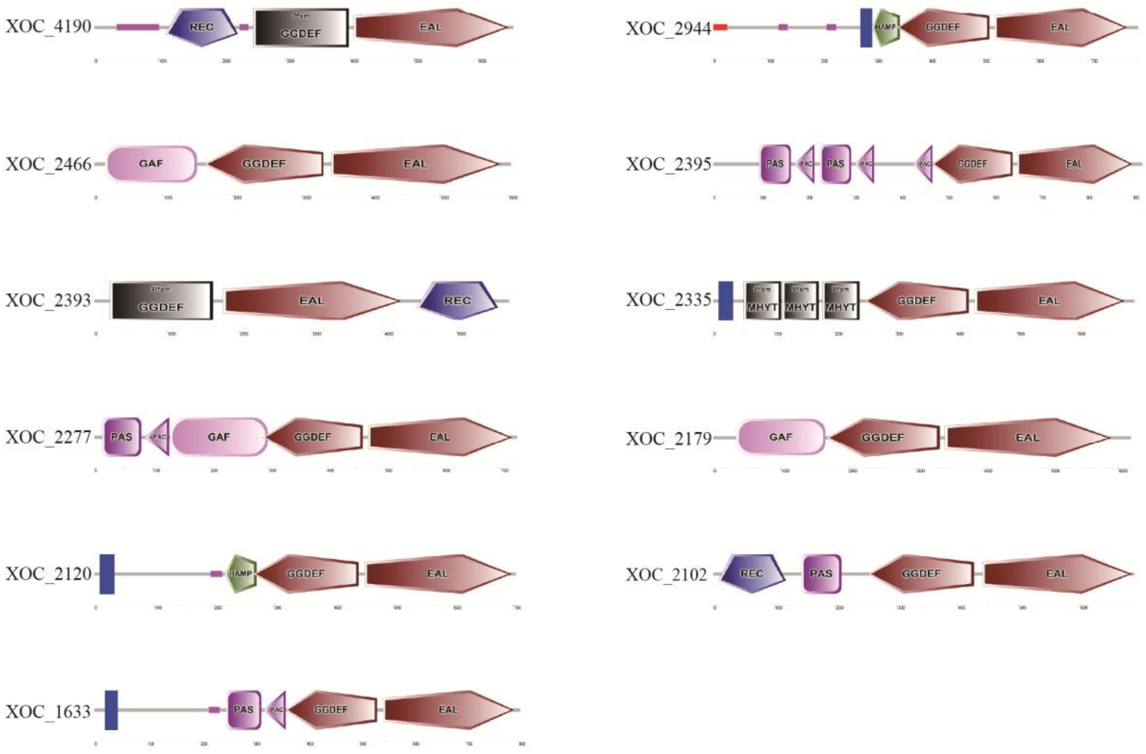

Supplementary Figure S1. Schematic representation of domain organization of 11 GGDEF-EAL containing proteins in *Xanthomonas oryzae* pv. *oryzicola*. The domain prediction was performed based on protein sequences derived from the genome sequences of *X. oryzae* pv. *oryzicola* BLS256 by the simplified modular architecture research tool (SMART) program. GGDEF, putative diguanylate cyclase domain; MHYT, NO-binding membrane sensor domain; REC, signal receiver domain; PAS, sensor domain for light and oxygen in signal transduction; GAF domain, related to small ligand binding and protein-protein interactions; EAL, putative phosphodiesterase domain; and HAMP, Histidine kinase, Adenylyl cyclase, Methyl-accepting protein domain.

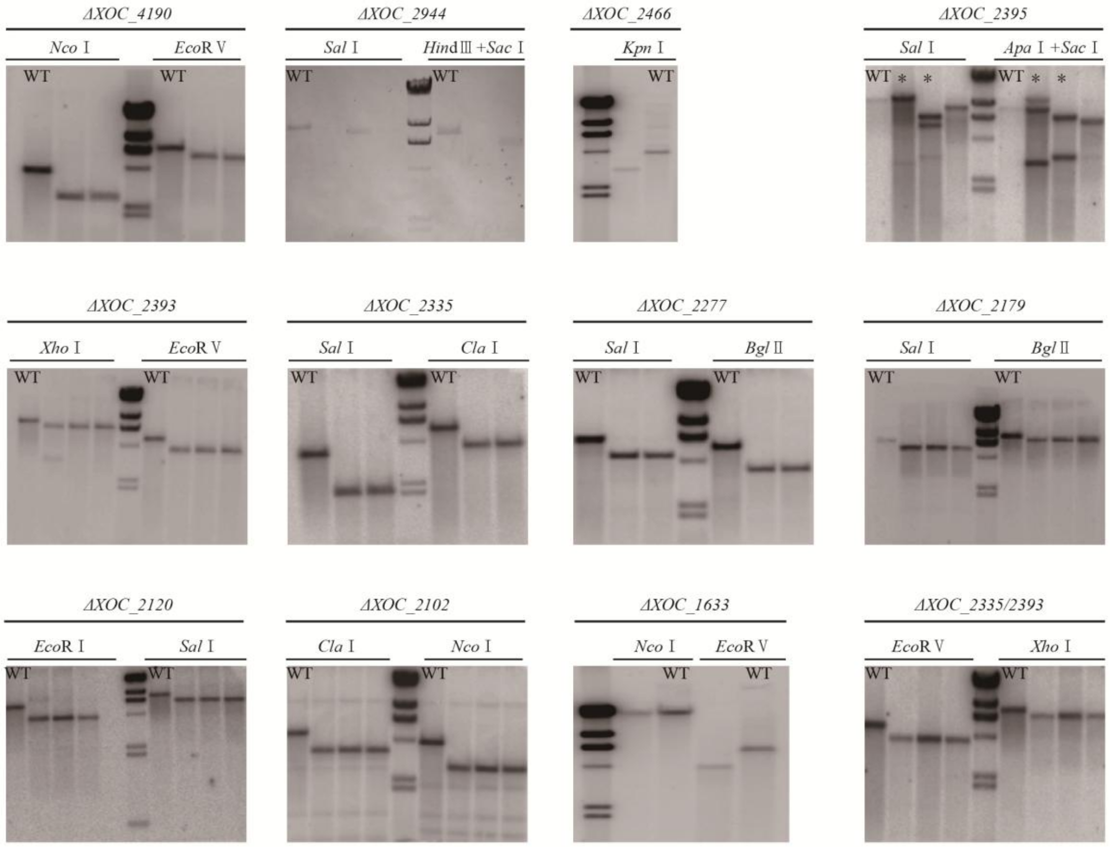

Supplementary Figure S2. The constructed non-marker *X. oryzae* pv. *oryzicola* mutants were confirmed by Southern blot analyses. The digested genomic DNA of the wild-type (WT) and mutant strains by the specified restriction enzymes was separated on agarose gel, blotted onto membranes and then probed with the isotope-labeled probes that were PCR amplified using the respective primer sets for each gene listed in Supplementary Table S1. * indicates the identified false gene-deletion mutants that were discarded. M: Molecular marker.

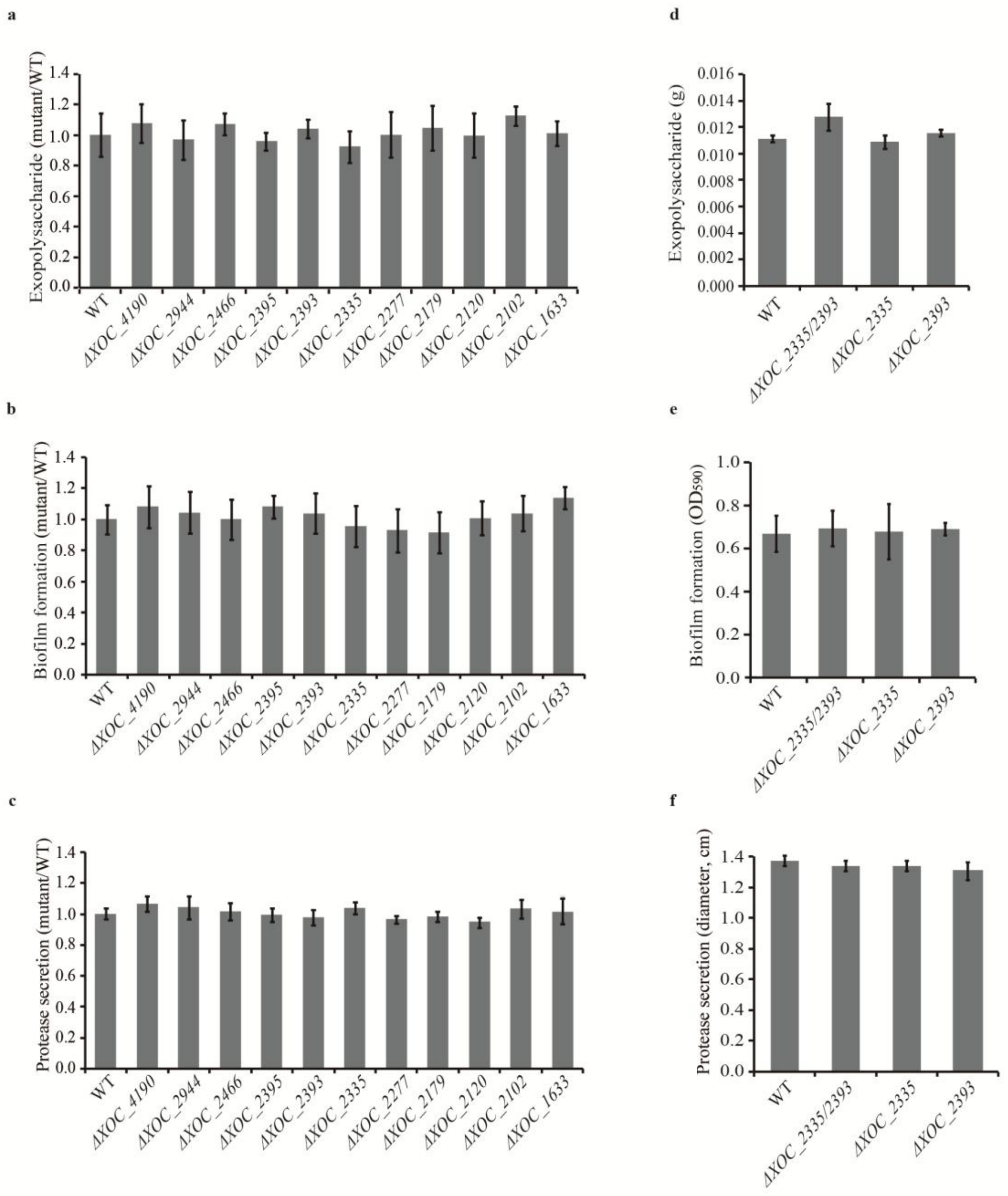

Supplementary Figure S3. Effects of mutations of genes encoding GGDEF-EAL domain-containing proteins on EPS production, biofilm formation and protease secretion in *X. oryzae* pv. *oryzicola*. All tested single-gene deletion mutants exhibited similar phenotypes in EPS production (a), biofilm formation (b) and protease secretion (c) to the wild type. The double-gene deletion mutant *ΔXOC_2335/XOC_2393* exhibited no significant alteration in EPS production (d), biofilm formation (e) and protease secretion (f) compared with the wild type.

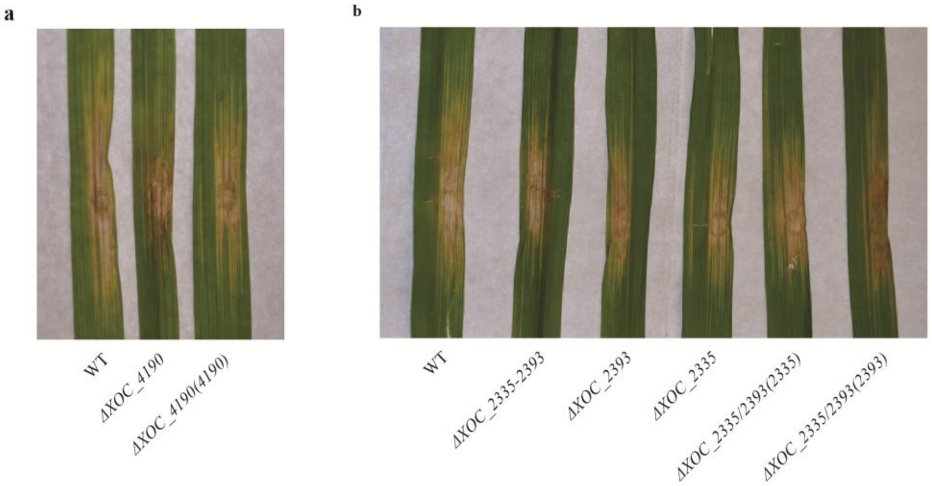

Supplementary Figure S4. Disease symptoms on the inoculated rice leaves caused by the *Xoc* wild-type, mutant and complementation strains. (a) Disease lesions on the leaves of rice cv. Shanyou 63 caused by pressure inoculation of the wild-type (WT), *ΔXOC_4190* and complemented strains, respectively. (b) Disease lesions on the inoculated leaves of rice cv. Shanyou 63 caused by *ΔXOC_2335*, *ΔXOC_2393*, the double-gene deletion mutant *ΔXOC_2335/XOC_2393* and complementation strains. The photographs were taken at 14 d after inoculation of rice leaves by different *Xoc* strains.

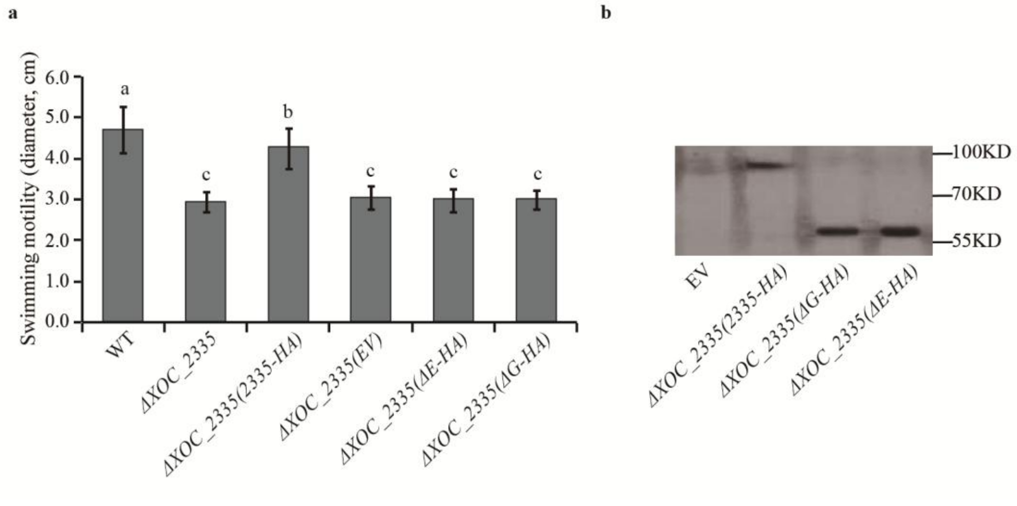

Supplementary Figure S5. The GGDEF or EAL domain of XOC_2335 alone is unable to complement the swimming motility of *ΔXOC_2335*. (a) The *XOC_2335(ΔG-HA)* or *XOC_2335(ΔE-HA)* construct did not complement the swimming motility of *ΔXOC_2335* while the *XOC_2335-HA* construct largely restored the swimming motility of *ΔXOC_2335* to the wild-type level. The colony diameters of different *Xoc* strains cultured on semisolid plates with 0.2% noble agar were shown. WT, the wild-type strain. (b) Expression of HA-tagged XOC_2335 and its truncated variants XOC_2335(ΔG-HA) and XOC_2335(ΔE-HA) in *Xoc* was detected by western blot analysis with an anti-HA antibody. EV indicates the wild-type strain transformed with the empty vector pVSP61 as negative control. The letters (a-c) indicate significant difference ($P < 0.05$) by Duncan's multiple range test.

Supplementary Table S1. Bacterial strains and plasmids

Strains/plasmids	Characteristics	Reference or source
E. coli		
DH5α	For high efficiency transformation	Lab collection
pRK600	Helper strain for tri-parental mating	Lab collection
BL21(DE3)	For protein expression	Lab collection
XL1-blue	For protein expression	Lab collection
X. oryzae* pv. *oryzicola		Lab collection
RS105	Wild-type, Rif^R	Lab collection
ΔXOC_4190	In frame deletion of *XOC_ 4190*, Rif^R	Lab collection
ΔXOC_2944	In frame deletion of *XOC_ 2944*, Rif^R	Lab collection
ΔXOC_2466	In frame deletion of *XOC_ 2466*, Rif^R	Lab collection
ΔXOC_2395	In frame deletion of *XOC_ 2395*, Rif^R	Lab collection
ΔXOC_2393	In frame deletion of *XOC_ 2393*, Rif^R	Lab collection
ΔXOC_2335	In frame deletion of *XOC_ 2335*, Rif^R	Lab collection
ΔXOC_2277	In frame deletion of *XOC_ 2277*, Rif^R	Lab collection
ΔXOC_2179	In frame deletion of *XOC_ 2179*, Rif^R	Lab collection
ΔXOC_2120	In frame deletion of *XOC_ 2120*, Rif^R	Lab collection
ΔXOC_2102	In frame deletion of *XOC_ 2102*, Rif^R	Lab collection
ΔXOC_1633	In frame deletion of *XOC_ 1633*, Rif^R	Lab collection
ΔXOC_2335/2393	In frame deletion of *ΔXOC_2335* and *ΔXOC_2393* Rif^R	This Study
ΔXOC_2335(2335)	Complementary strain, pVSP61-*XOC_2335* transformed into *ΔXOC_2335*, Rif^R Km^R	This Study
ΔXOC_4190(4190)	Complementary strain, pVSP61-*XOC_4190* transformed into *ΔXOC_4190*, Rif^R Km^R	This study
ΔXOC_2393(2393)	Complementary strain, pVSP61-*XOC_2393* transformed into *ΔXOC_2393*, Rif^R Km^R	This study
ΔXOC_2102(2102)	Complementary strain, pVSP61-*XOC_2102* transformed into *ΔXOC_2102*, Rif^R Km^R	This study
ΔXOC_2335/2393(2335)	Complementary strain, pVSP61-*XOC_2335* transformed into *ΔXOC_2335/2393*, Rif^R Km^R	This study
ΔXOC_2335/2393(2393)	Complementary strain, pVSP61-*XOC_2393* transformed into *ΔXOC_2335/2393*, Rif^R Km^R	This study
**Plasmids**		
pUFR80	Suicide vector for homologous recombination, Km^R	Lab collection
pVSP61	Expression plasmid for complementation, Km^R	Lab collection
pQE30	*In vitro* protein expression vector, Amp^R	Lab collection

pET28a	*In vitro* protein expression vector, Km^R	Lab collection
pVSP61-*XOC_2335*	Complementation, *XOC_2335* cloned in pVSP61, Km^R	This study
pVSP61-*XOC_4190*	Complementation, *XOC_4190* cloned in pVSP61, Km^R	This study
pVSP61-*XOC_2393*	Complementation, *XOC_2393* cloned in pVSP61, Km^R	This study
pVSP61-*XOC_2102*	Complementation, *XOC_2102* cloned in pVSP61, Km^R	This study
pVSP61-*XOC_2335(2335-HA)*	Complementation, *XOC_2335-HA* cloned in pVSP61, Km^R	This study
pVSP61-*XOC_2335(EAA-HA)*	Complementation, *XOC_2335-EAA-HA* cloned in pVSP61, Km^R	This study
pVSP61-*XOC_2335(GAAAF-HA)*	Complementation, *XOC_2335-GAAAF-HA* cloned in pVSP61, Km^R	This study
pVSP61-*XOC_2335(ΔE)*	Complementation, *XOC_2335-ΔE-HA* cloned in pVSP61, Km^R	This study
pVSP61-*XOC_2335(ΔG)*	Complementation, *XOC_2335-ΔG-HA* cloned in pVSP61, Km^R	This study
pQE30-*4190*	*XOC_4190* cloned in pQE30, Amp^R	This study
pQE30-*2102*	*XOC_2102* cloned in pQE30, Amp^R	This study
pET28a-*4190ΔG*	*XOC_4190ΔG* cloned in pET28a , Km^R	This study
pET28a-*4190ΔE*	*XOC_4190ΔE* cloned in pET28a , Km^R	This study

Supplementary Table S2. Primers used in this study

Primer names	Primer sequences (5'→3')
	**Primers for gene deletion**
XOC_2466-*Bam*HI-F	ATGGATCCTGGCATTGGGCGGGTTTAGCG
XOC_2466-del-R	TTTCGTCGGCATGCTCGATGCAATTCCAACTGTGCGGCAG
XOC_2466-del-F	CTGCCGCACAGTTGGAATTGCATCGAGCATGCCGACGAAA
XOC_2466-*Hin*dIII-R	TTAAGCTTGTACGACCAGGCCGGCATCAT
XOC_2277-*Bam*HI-F	ATGGATCCTGCCCGGTCACGCATAC
XOC_2277-del-R	CGCGCGTCTTCGCTGTGTTCGATAGACCAGCAAGTCGGCG
XOC_2277-del-F	CGCCGACTTGCTGGTCTATCGAACACAGCGAAGACGCGCG
XOC_2277-*Sal*I-R	AAGTCGACGCGCGCAACCATTTGAGGCA
XOC_2944-*Bam*HI-F	AAGGATCCAAGGCATCACTGCTGCGCC
XOC_2944-del-R	GAGCGCGGACAGATTGATTGCGCCTGTTGCAGCAATGCCT
XOC_2944-del-F	AGGCATTGCTGCAACAGGCGCAATCAATCTGTCCGCGCTC
XOC_2944-*Hin*dIII-R	TTAAGCTTGGCGCCAAACACGTCCTGAC
XOC_4190-*Bam*HI-F	AGGATCCGGGTCTATCTCACCGTGTTC
XOC_4190-del-R	GCAAGCCCAATGCGTGGTCCATCGTCAGAGGTCGCGGCAG
XOC_4190-del-F	CTGCCGCGACCTCTGACGATGGACCACGCATTGGGCTTGC
XOC_4190-*Hin*dIII-R	TTAAGCTTCGGCAGCCTGTTTGGCGC
XOC_2120-*Sal*I-F	ATTGTCGACTTCCACACGCTGTTCTTCG
XOC_2120-del-R	GTCGATCTTGACCCCGTCCACCAGCACATAGCTCACCACC
XOC_2120-del-F	GGTGGTGAGCTATGTGCTGGTGGACGGGGTCAAGATCGAC
XOC_2120-*Eco*RI-R	TGAATTCGACTCCGCAGTGGACA
XOC_2335-*Bam*HI-F	AAGGATCCACGAAGCGCTCAGCCTGG
XOC_2335-del-R	GGCGACTACCTGCAGACGCATGCATCAGCGCAATGCCCAG
XOC_2335-del-F	CTGGGCATTGCGCTGATGCATGCGTCTGCAGGTAGTCGCC
XOC_2335-*Hin*dIII-R	TTAAGCTTGGTGAGGTCGGGCATGAT
XOC_2179-*Sal*I-F	AAAGTCGACACGCATTGGTCGCATCAGC
XOC_2179-del-R	ATTCGATTTCCAGCCGGCGCGGATCGCGTGCGTGCAGAAC
XOC_2179-del-F	GTTCTGCACGCACGCGATCCGCGCCGGCTGGAAATCGAAT
XOC_2179-*Hin*dIII-R	TTAAGCTTCAAGATCGGCGCGGCC
XOC_2393-*Eco*RI-F	ATGAATTCTCGTTGCAGGAAGACGAACG
XOC_2393-del-R	CGACGCAGGAAGCCCAATTGCGAAGTGATCGATGTCGACG
XOC_2393-del-F	CGTCGACATCGATCACTTCGCAATTGGGCTTCCTGCGTCG
XOC_2393-*Hin*dIII-R	TTAAGCTTCGACCCGTTTTTCACCACCA
XOC_1633-*Bam*HI-F	TGGATCCTGGATCTGCCGGCCGACAT
XOC_1633-del-R	TGATCTCCATGCACAGCTTCGGCAAGCGGCTTGCAGATCGG
XOC_1633-del-F	CCGATCTGCAAGCCGCTTGCCGAAGCTGTGCATGGAGATCA
XOC_1633-*Hin*dIII-R	TTAAGCTTGTTGAGCGCGATGTAGACA
XOC_2102-*Bam*HI-F	AGCTGGATCCGCCGCAGTTGCAG
XOC_2102-del-R	TCTGGCGTCTGCAGCCACAGCCATGCGAAGCGGCCTCGAC
XOC_2102-del-F	GTCGAGGCCGCTTCGCATGGCTGTGGCTGCAGACGCCAGA
XOC_2102-*Hin*dIII-R	TTAAGCTTCGGCATGTTCGCGACCATGAT
XOC_2395-*Kpn*I-F	AAGGTACCGGATTCGCGTCGAGGATGC

XOC_2395-del-R	CGAACAGTTCGAAGCAGCGGCTCGGGAATGCTCAAGGTGA
XOC_2395-del-F	TCACCTTGAGCATTCCCGAGCCGCTGCTTCGAACTGTTCG
XOC_2395-*Hin*dIII-R	TAAAGCTTATCGCGTGACACGGGTACG
	**Primers of probes for Southern blot**
2102-F	CCGAAAGGGATCATTCACCCT
2102-R	CTTGGACATCGCCTTGAGCAG
2120-F	GGCGCCTGCATTGAAACGT
2120-R	CGCCACGTTCGATCTGCTT
2179-F	ACGATTGCTCGGGAAGGCA
2179-R	AGCCAGGTATCCAGGTCTTCG
2277-F	CCCGACGCTTCCACTGACA
2277-R	CGATCTTCAGGCCGTTGACC
2335-F	TCGCTAAGTCTTGGGCAACGC
2335-R	CCAGCGATTGCATCAGCACG
2393-F	GAGCCTCACGAATGCGTTGC
2393-R	TCTTCGGCATAGTCCAGGAGC
2395-F	TGCAGCCATCTTAGCCGTAACTC
2395-R	GGCAATGCGTTGGGCTTCT
2944-F	CGTAACGCCGACCCGAAGA
2944-R	CGCTTGCAGCCTGACCAGAT
4190-F	GCAGGCGCTAGAAATCCGTCA
4190-R	CGGATCTTCGATGCGTTGGC
1633-F	CATACAGACTGAGCCGTTGCG
1633-R	CCTTCGTGCGACAGCAGGTAAT
2466-F	GCGTGGCATCAACAGCAG
2466-R	CGCATCGCATCGTTGGTGT
	**Primers for constructing complementation strains**
C4190-*Bam*HI-F	AGGATCCGGGTCTATCTCACCGTGTTC
C4190-*Hin*dIII-R	TTAAGCTTCGGCAGCCTGTTTGGCGC
C2335-*Bam*HI-F	AAGGATCCACGAAGCGCTCAGCCTGG
C2335-*Hin*dIII-R	TTAAGCTTGGTGAGGTCGGGCATGAT
C2102-*Bam*HI-F	AGCTGGATCCGCCGCAGTTGCAG
C2102-*Hin*dIII-R	TTAAGCTTCGGCATGTTCGCGACCATGAT
C2393-*Eco*RI-F	ATGAATTCTCGTTGCAGGAAGACGAACG
C2393-*Hin*dIII-R	TTAAGCTTCGACCCGTTTTTCACCACCA
	**Primers for site-directed mutagenesis**
2335-EAA-F	CGATTGGCGCTGAAGCAGCAATCCGCTGGCAG
2335-EAA-R	CTGCCAGCGGATTGCTGCTGCTTCAGCGCCAATCG
2335-GAAAF-F	GGCTGGGCGCAGCCGCATTCGTGCTGGTGGC
2335-GAAAF-R	GCCACCAGCACGAATGCGGCTGCGCCCAGCC
	**Primers for constructing C-terminal HA-tagged *XOC_2335***
2335-*Sac*I-*HA*-F	GCACGAGCTCAAGATCGACCGCAGC
2335-*Hin*dIII-*HA*-R	TAAAGCTTACGCATAGTCAGGAACATCGTATGGG
	TAGCTAGCCAGGCGGCGCGTGCCGT

2335-Δ*E*- *Hin*dIII-*HA*-R	TAAAGCTT ACG CAT AGT CAG GAA CAT CGT ATG GGT
2335-Δ*G*-F	AGA AAA AGC TGT AGC GGT TGC GGC
2335-Δ*G*-R	AGC AGC TGG TGC TCG CTA CCG CCC GTC AAC GCC AGG TCA T
	**Primers for constructing protein expression vectors**
pQE30-*4190* -*Bam*HI-F	TAGGATCCATGGCCGCCCGAGATCCGGTT
pQE30-*4190* -*Hin*dIII-R	TAAAGCTTCAGAGCACCGCGTTCGTGAAATC
pET28a-*4190*Δ*G*-*Bam*HI-F	AAGGATCCAGCCAGGCAGAACAGCAGGAAC
pET28a-*4190*Δ*E*-*Hin*dIII-R	TAAAGCTTGGG TGC CAG ATA GGT CTG CAC
pQE30-*2102*-*Bam*HI-F	AAGGATCCATGCAAAAAGGCAAAGATCTCACC
pQE30-*2102* -*Hin*dIII-R	ATAAGCTTCAGCCGAACTCGTAGTTCATC
	**Primers for quantificational real-time polymerase chain reaction**
16S-qRT-F	CGCAAGACTGAAACTCAAAGG
16S-qRT-R	AAGGCACCAATCCATCTCTG
hrpA-qRT-F	ATCAAGTTCATGACCGACGG
hrpA-qRT-R	ACTGTTTGAGATAGCCCAGC
hrpG-qRT-F	GAGTTACTGGTCTTCGACGC
hrpG-qRT-R	CCAGTCCAGGATGTCATTGG
hrpX-qRT-F	TCCTTTCGACCTACTTTGCAG
hrpX-qRT-R	TCGCTGTTGAAGGTGCTG
